# Development and validation of a novel HPLC-UV method for simultaneous determination of azathioprine metabolites in human red blood cells

**DOI:** 10.1016/j.heliyon.2023.e13870

**Published:** 2023-02-21

**Authors:** Hengyi Yu, Dongyan Li, Dong Xiang, Xiping Li, Lu Liu, Dong Liu, Xuepeng Gong

**Affiliations:** Department of Pharmacy, Tongji Hospital, Tongji Medical College, Huazhong University of Science and Technology, Hubei Wuhan 430030, China

**Keywords:** HPLC-UV, Inflammatory bowel disease, Azathioprine, Therapeutic drug monitoring

## Abstract

A rapid, specific and accurate high-performance liquid chromatography with tunable ultraviolet detection method was developed to simultaneously determine azathioprine metabolites, 6-thioguanine nucleotides (6-TGN) and 6-methyl mercaptopurine riboside (6-MMPr) in human red blood cells. Erythrocyte lysate sample was precipitated by perchloric acid under the protection of dithiothreitol, with 6-TGN and 6-MMPr being acid hydrolyzed to produce 6-thioguanine (6-TG) and 6-methymercaptopurine (6-MMP). A Waters Cortecs C_18_ column (2.1 × 150 mm, 2.7 μm) was used for chromatographic separation with a water (containing 0.01 mol/L ammonium acetate and 0.2% acetic acid)/methanol linear gradient at a flow rate of 0.45 mL/min in a 5.5 min. UV detection wavelengths were 340 nm for 6-TG, 303 nm for 6-MMP and the IS (5-bromouracil). The calibration curves fitted a least squares model (weighed 1/*x*^2^) from 0.15 to 15 μmol/L for 6-TG (*r*^*2*^ = 0.9999) and from 1 to 100 μmol/L for 6-MMP (*r*^*2*^ = 0.9998). This method was validated according to the FDA bioanalytical method validation guidance and ICH M10 bioanalytical method validation and study sample analysis guidance for industry, and successfully utilized in ten IBD patients receiving azathioprine therapy.

## Highlights

• A novel HPLC-UV method developed for simultaneous determination of azathioprine metabolites.
• Short turn-around time (5.5 min) allowed for a high throughput analysis at a relatively low cost.
• Successfully applied to therapeutic drug monitoring for azathioprine in IBD patients.

## 1 Introduction

Inflammatory bowel diseases (IBD), primarily ulcerative colitis and Crohn's disease, are autoimmune disorders with the characteristic intermittent course and manifested by chronic gastrointestinal inflammation [1]. At present, aminosalicylates, steroids, immunomodulators, and biologics are current durg therapies used for IBD according to the site of disease and disease activity [2], and therapeutic drug monitoring of thiopurines and biologics was encouraged in IBD patients [3,4].

Thiopurines (i.e. azathioprine and mercaptopurine) are an inexpensive and effective treatment option to maintain remission in the majority of IBD patients [5]. Although the role of thiopurines has been doubted in recent years because of their toxicities and increasing choice of targeted therapies available [6], thiopurines remain an attractive option in the right patients owing to their low cost [7]. As the main thiopurine used for the treatment of IBD in China [8], azathioprine is a precursor drug with complex metabolism [9]. After being absorbed, azathioprine is rapidly converted to 6-mercaptopurine (6-MP) by glutathione S-transferase (GST), which is further metabolized to different thiopurine nucleotides (TPNs) by a series of enzymes, including hypoxanthine guanine phosphoribosyltransferase (HPRT), xanthine oxidase (XO), and thiopurine S-methyltransferase (TPMT) [9]. 6-Thioguanine nucleotides (6-TGN, therapeutic window 235–450 pmol/8 × 10^8^RBC) [10] is the main active metabolite of azathioprine formed by HPRT pathway that produces therapeutic effect by integrating cellular DNA into nonfunctional nucleotides and nucleic acids, but is also the main cause of bone marrow toxicity [[11], [12], [13]]. Azathioprine can also be transformed into 6-methylmercaptopurine riboside (6-MMPr, normal value < 5799 pmol/8 × 10^8^RBC) [10] through TPMT pathway, and the erythrocyte concentration of 6-MMPr was considered to be related to hepatotoxicity closely and to competitively inhibit the production of 6-TGN [14]. Therefore, monitoring the concentrations of 6-TGN and 6-MMPr in red blood cells is becoming integrated in general IBD practice for optimizing efficacy and minimizing toxicity [3,4].

As shown in Table 1, various analytical methods were reported for determination of 6-TGN and 6-MMPr in red blood cell [15], [16], [17], [18], [20], [21], [22], [23], [25] or whole blood [18,19,24,[26], [27], [28]] in patients receiving azathioprine treatment. As the most common practice at present, erythrocyte sample was precipitated by perchloric acid under the protection of dithiothreitol (DTT), with 6-TGN and 6-MMPr being acid hydrolyzed to yield 6-thioguanine (6-TG) and 6-methymercaptopurine (6-MMP) and detected by LC-UV [[15], [16], [17], [18], [19], [20]] or LC-MS/MS methods [[21], [22], [23], [24], [25], [26], [27], [28]]. However, the existing LC-UV methods often need long run time (most ≥10 min) and large volume of mobile phase solvent. Although the LC-MS/MS assays are faster and more sensitive than LC-UV methods, this kind of equipment is too expensive for the majority of laboratories in developing countries, and obvious matrix effects were reported in many LC-MS/MS methods [21,26]. Besides, perchloric acid is another problem in LC-MS/MS methods, which could contaminate the ion-source and influence the signals of other compounds on the same instrument, both reported in references [21,23] and observed in our laboratory. In this work, we developed a novel HPLC-UV method that could provide short turn-around analysis time at a relatively low cost for simultaneous determination of azathioprine metabolites 6-TGN and 6-MMPr.

**Table 1:** Summary of the reported LC-UV and LC-MS methods for azathioprine metabolites.

Method	Year	Matrix	Matrix volume (μL)	Analyte	IS	IS normalized matrix factor (%)	Linear range	Flow rate (mL/min)	Analytical time (min)	Reference
HPLC-UV	1992	erythrocyte	200	6-TG	no	not available	30-900 pmol/8 × 10^8^ RBC	not mentioned	10.0	[15]
				6-MMP	no		300-30000 pmol/8 × 10^8^ RBC			
	1998	erythrocyte	500	6-TG	no	not available	0.3–50 μmol/L	1.20	12.0	[16]
				6-MMP	no		1.5–120 μmol/L			
	2009	erythrocyte	200	6-TG	no	not available	30-1500 pmol/8 × 10^8^ RBC	1.00	13.0	[17]
				6-MMP	no		250-24,000 pmol/8 × 10^8^ RBC			
	2012	erythrocyte/whole blood	500	6-TG	5-bromouracil	not available	50-1600 pmol/8 × 10^8^ RBC	1.00	20.0	[18]
				6-MMP	5-bromouracil		375-12000 pmol/8 × 10^8^ RBC			
	2015	whole blood	500	6-TG	5-bromouracil	not available	50-2000 pmol/8 × 10^8^ RBC	0.60	7.5	[19]
				6-MMP	5-bromouracil		20-15000 pmol/8 × 10^8^ RBC			
	2021	erythrocyte	200	6-TG	caffeine	not available	146-4877 pmol/8 × 10^8^ RBC	1.00	31.0	[20]
				6-MMP	caffeine		147-4906 pmol/8 × 10^8^ RBC			
HPLC-MS/MS	2005	erythrocyte	100	6-TG	8-bromoadenine	63	0.25–20 μmol/L	0.30	8.0	[21]
				6-MMP	8-bromoadenine	100	2.5–200 μmol/L			
	2017	erythrocyte	100	6-TG	methyl 9H-pyrido [3,4-b] indole-3-carboxylate	103–149	0.1–20 μmol/L	0.60	6.5	[22]
				6-MMP	methyl 9H-pyrido [3,4-b] indole-3-carboxylate	83–115	0.1–20 μmol/L			
	2019	erythrocyte	250	6-TG	6 TG-^13^C_2_^15^N	103–104	9.2–1400 pmol/8 × 10^8^ RBC	1.00	5.0	[23]
				6-MMP	6MMP-d_3_	86–102	179-28000 pmol/8 × 10^8^ RBC			
	2020	whole blood (dried blood spot)	15	6-TG	8-bromoadenosine	not mentioned	0.5–15 μmol/L	0.3	10.0	[24]
				6-MMP	8-bromoadenosine	not mentioned	3.75–175 μmol/L			
	2022	erythrocyte	100	6-TG	6 TG-^13^C_2_^15^N	not mentioned	0.2–7.5 μmol/L	0.4	7.0	[25]
				6-MMP	6MMP-d_3_	not mentioned	4-150 μmol/L			
UPLC-MS/MS	2013	whole blood	25	6-TG	6 TG-^13^C_2_^15^N	81–146	30-10000 pmol/0.2 mL	1.00	5.1	[26]
				6-MMP	6MMP-d_3_	74–114	30-10000 pmol/0.2 mL			
	2016	whole blood	200	6-TG	6 TG-^13^C_2_^15^N_2_	not mentioned	50-1700 ng/mL	0.50	3.0	[27]
				6-MMP	6 TG-^13^C_2_^15^N_2_	not mentioned	500-30000 ng/mL			
	2020	whole blood	200	6-TG	6 TG-^13^C_2_^15^N	95–101	1.25 to 5000 ng/mL	0.40	2.0	[28]
				6-MMP	6 TG-^13^C_2_^15^N	92–110	1.25 to 5000 ng/mL			
this method		erythrocyte	100	6-TG	5-bromouracil	not available	0.15–15 μmol/L	0.45	5.5	
				6-MMP	5-bromouracil		1-100 μmol/L			

## 2 Materials and methods

### 2.1 Chemicals and reagent

**Fig. 1:**
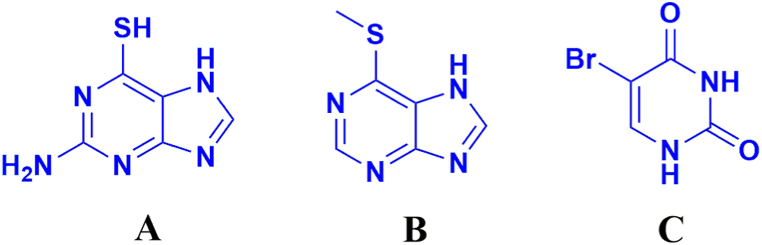
Chemical structures of 6-TG (A), 6-MMP (B) and the internal standard 5-bromouracil (C).

6-Thioguanine (6-TG) was purchased from National Institutes for Food and Drug Control (Beijing, China). 6-methylmercaptopurine (6-MMP) was obtained from Shanghai Yien Chemical Technology Co., Ltd (Shanghai, China). 5-Bromouracil was purchased from Macklin (Shanghai, China) as the internal standard (IS). Dl-dithiothreitol (DTT), along with HPLC grade of acetic acid, formic acid, phosphoric acid, ammonium acetate, ammonium formate and potassium dihydrogen phosphate were all supplied from Aladdin Industry Corporation (Shanghai, China). HPLC grade of methanol and acetonitrile were obtained from Tedia (USA). Deionized water was prepared by Elga Purelab Felx 3 water purification system (ELGA, High Wycombe, UK). Blank human blood from healthy volunteers were obtained from Phase I Clinical Laboratory of Tongji Hospital. The structures of analytes were shown in Fig. 1.

### 2.2 Instrumentation and chromatographic conditions

Waters ACQUITY UPLC H-Class system (Milford, MA, USA) consisting of a quaternary solvent manager, a column heater, a sample manager, and a tunable ultraviolet (TUV) detector was used, with the Waters Empower™ version 3.0 software (Milford, MA, USA) for data acquisition and processing.

A Waters Cortecs C_18_ column (2.1 × 150 mm, 2.7 μm) was used for chromatographic separation. Solution A consisted of 0.01 mol/L ammonium acetate and 0.2% acetic acid (v/v) in water, and solution B was methanol. The flow rate was maintained at 0.45 mL/min and the injection volume was 10 μL. A gradient program in 5.5 min was performed as follows: 0−2 min 5% B, 2−3 min 5−20% B, 3.01−4.0 min 60% B, 4.01−5.5 min 5% B. The monitoring wavelengths were set at 340 nm for 6-TG, 303 nm for 6-MMP and 5-bromouracil (IS) [16].

### 2.3 Stock solutions, standard and quality control (QC) solutions/samples

The stock solution of 6-TG, 6-MMP and 5-bromouracil were prepared in 50 mL volumetric flasks at a concentration of about 0.3 mg/mL, with water containing 0.1 mol/L NaOH. The stock solutions of 6-TG and 6-MMP were further diluted with water containing 0.1 mol/L NaOH and mixed to obtain the standard solutions and QC solutions of two analytes at several concentrations. The stock solution of 5-bromouracil was diluted to 576 μmol/L in water containing 0.1 mol/L NaOH as the IS working solution. The stock, standard and QC solutions of two analytes along with the IS stock/working solutions were all stored at −80 °C.

The calibration curves were established through eight calibration standards prepared by adding 20 μL of standard solution to 280 μL of blank erythrocyte lysate, giving the final concentrations of 0.15, 0.3, 0.75, 1.5, 3.75, 7.5, 12, 15 μmol/L for 6-TG, and 1, 2, 5, 10, 25, 50, 80, 100 μmol/L for 6-MMP. QC samples were prepared at four concentration levels for final concentrations of 0.15, 0.45, 3 and 11.25 μmol/L for 6-TG, and 1, 3, 20 and 75 μmol/L for 6-MMP.

### 2.4 Sample preparation

About 2 mL of the whole blood was collected using an EDTA tube from patient receiving azathioprine. Hematocrit (HCT) and erythrocyte counts (RBC) were obtained from each sample. After centrifugation (2000 g, 4 °C, 5 min) to separate plasma and buffy coat, the erythrocytes were washed with approximately 2 mL of saline and packed by centrifugation (2000 g, 4 °C, 5 min) twice. To obtain the erythrocyte lysate, 100 μL of erythrocyte and 200 μL of deionized water was transferred to a 2 mL centrifugal tube and mixed for 1 min. The erythrocyte lysate would be stored at −80 °C and analyzed within 7 days.

For analysis, 20 μL of the IS working solution (576 μmol/L) and 60 μL of water-dissolved DTT (0.5 mol/L) were added to the 2 mL centrifugal tube containing 300 μL of erythrocyte lysate and mixed for 1 min, after which 40 μL of 70% perchloric acid was added and mixed for 1 min. The mixture was then centrifuged (12000 g, 4 °C, 10 min) to obtain the supernatant. After being transferred to a 0.5 mL centrifugal tube, 300 μL of the supernatant was heated for 45 min in an electric bath at 100 °C. After being cooled down and centrifuged (12000 g, 4 °C, 10 min), 200 μL of the supernatant was transferred to a vial and 10 μL was injected for analyzing. The concentrations of 6-TG and 6-MMP were presented in μmol/L units, which could be converted into pmol/8 × 10^8^RBC by the formula below: $C_{pmol/8\times 10^{8}RBC}=2400\times C_{\mu mol/L}\times HCT/RBC$, where $C_{pmol/8\times 10^{8}RBC}$ stands for concentration in pmol/8 × 10^8^RBC, $C_{\mu mol/L}$ stands for concentration in μmol/L, HCT stands for hematocrit, and RBC stands for red blood cell count in 10^12^/L.

### 2.5 Method validation

The method was validated according to the FDA bioanalytical method validation guidance for industry [29] and ICH M10 bioanalytical method validation and study sample analysis guidance for industry [30].

#### 2.5.1 Selectivity

Endogenous and exogenous interferences were examined through analyzing blank human erythrocyte lysate samples from six individual sources and comparing the chromatograms with the corresponding spiked erythrocyte lysate samples at lower limit of quantification (LLOQ) level. When the response of interference is below 20% of the LLOQ and 5% for the IS, the interfering substances were thought to be absent. The LLOQ for each analyte is the lowest nonzero standard on the calibration curve, and the analyte response of LLOQ should be ≥ five times the analyte response of the zero calibrator.

#### 2.5.2 Carryover

Carryover was examined by injecting blank erythrocyte lysate samples after the highest standard in the calibration curve. Carryover was considered acceptable if the response in the blank erythrocyte lysate sample is ≤ 20% of the response of the LLOQ.

#### 2.5.3 Calibration curve and range

Calibration curves for 6-TG and 6-MMP were established through eight calibration standards in three days and processed by weighed (1/*x*^2^) least-squares linear regression analysis.

#### 2.5.4 Accuracy and precision

The accuracy and precision were evaluated by analyzing QC samples at four concentration levels. The RE (relative error of the nominal and measured values) and CV (coefficient of variation) for the within-run assays were examined based on the analysis of six samples. The RE and CV for the between-run assays were assessed at the same concentration and repeated in three days. The RE between the measured and nominal concentrations of the standards should be within ±15%, and ±20% is acceptable for LLOQ.

#### 2.5.5 Recovery

The recovery was assessed at low, medium, and high QC levels in six replicates. Extraction recovery of 6-TG and 6-MMP was determined by comparing the peak areas of six extracted spiked samples with the mean peak area of six post-extracted samples. The recovery of the IS was also assessed.

#### 2.5.6 Stability

In our hospital, the whole blood samples were stored at 4 °C immediately after collection from patients, transferred to the pharmacological laboratory within 24 h, and analyzed with 7 days. Based on the above practice, three replicates of QC samples at low and high levels were used to evaluate the stability of 6-TG and 6-MMP in erythrocytes lysate under the following conditions: 4 °C for 24 h, 25 °C for 4 h, −80 °C for 7 d, three freeze-thaw cycles from −80 °C to 25 °C, and post-treatment in the autosampler at 25 °C for 24 h. Additionally, stock solution stability at −80 °C for 30 days was also evaluated. Analyte was deemed to be stable when the RE between the fresh sample and the stability testing sample were within ±15%.

### 2.6 Determination of clinical samples

Samples obtained from ten IBD patients receiving azathioprine were analyzed through the validated HPLC-UV method. Approximately 2 mL of blood was collected using an EDTA tube and stored at 4 °C immediately. The blood samples were then transferred to the pharmacological laboratory within 24 h and prepared as erythrocyte lysate for analysis.

## 3 Results and discussion

### 3.1 Method development

At present, several LC-UV and LC-MS/MS methods have been developed for simultaneous determining 6-TGN and 6-MMPr in erythrocyte or whole blood (Table 1). However, these methods are limited by the long run time (LC-UV) or expensive instrument cost (LC-MS/MS), which inspired us to develop a novel HPLC-UV method that could provide fast analysis time at a relatively low cost.

To improve the separation performance, we compared several chromatographic columns, such as Waters Cortecs C_18_ column (2.1 × 150 mm, 2.7 μm), Waters ACQUITY UPLC BEH C_18_ column (2.1 × 50 mm, 1.7 μm), Waters Symmetry C_18_ column (2.1 × 150 mm, 3.5 μm) and Dikma Leapsil C_18_ column (2.1 × 150 mm, 2.7 μm). Finally, Waters Cortecs C_18_ column (2.1 × 150 mm, 2.7 μm) was selected owing to the symmetrical peak shape and optimal peak separation from endogenous and exogenous interference.

In terms of the organic mobile phase, methanol could result in better peak separation between 6-TG and the interference than that of acetonitrile. Meanwhile, water containing 0.01 mol/L ammonium acetate and 0.2% acetic acid was chosen as the aqueous mobile phase, resulting in better peak separation between 6-MMP/IS and the interferences than those of water containing ammonium formate/formic acid, or water containing potassium dihydrogen phosphate/phosphoric acid. Under the optimized chromatographic condition, 6-TG, 5-bromouracil and 6-MMP were successfully separated in 5.5 min, with the retention times of 1.5, 1.7 and 4.0 min, respectively.

In terms of the injection volume, the analyte response of 6-TG and 6-MMP increased with the increasing of injection volume, while the peak symmetry was not influenced. Finally, the largest injection volume of 10 μL was selected.

As mentioned above, 6-TGN was acid hydrolyzed into 6-TG for analysis. Because the sulfhydryl group in 6-TGN or 6-TG was apt to be oxidized, DTT was added to protect the sulfhydryl group. It was reported that increasing DTT concentration could enhance the recovery of 6-TG [31], and 60 μL of 0.5 mol/L DTT was found to yield higher recovery of 6-TG in this method.

Based on the literature [21], we also compared the peak areas of the samples after being heated at 100 °C for 45 min and 60 min. As a result, no significant difference existed between 45 min and 60 min.

### 3.2 Method validation

#### 3.2.1 Selectivity and carryover

**Fig. 2:**
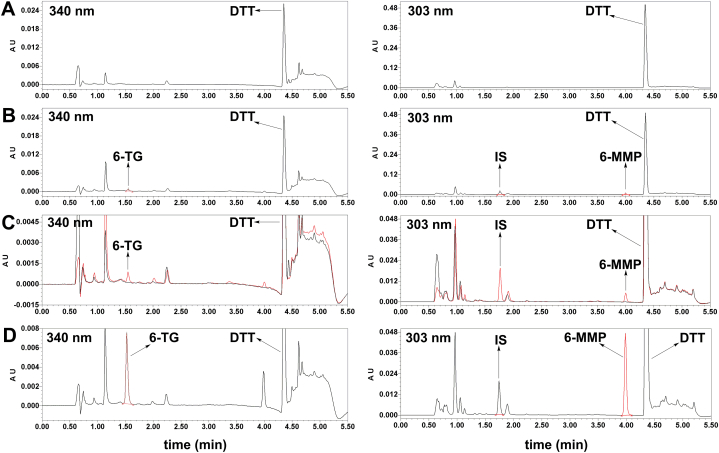
Full chromatograms of blank erythrocyte lysate (A) and blank erythrocyte lysate spiked with LLOQs (B). Partial enlarged chromatograms of blank erythrocyte lysate (grey lines) and QC sample at the LLOQ level (red lines) (C), and samples from IBD patients undergoing azathioprine treatment (D). (For interpretation of the references to colour in this figure legend, the reader is referred to the Web version of this article.)

Typical chromatograms for the blank erythrocyte lysate [Fig. 2(A)], blank erythrocyte lysate spiked with LLOQs for 6-TG and 6-MMP [Fig. 2(B–C)], and erythrocyte lysate from patients receiving azathioprine therapy [Fig. 2(D)] are presented in Fig. 2. The peak shapes of 6-TG, 5-bromouracil (IS) and 6-MMP were very good at retention time of 1.5 min, 1.7 min and 4.0 min, respectively. No obvious interfering substances existed in the chromatograms of blank erythrocyte lysate and sample from patients. Remarkably, the total run time was 5.5 min per sample, wihch was shorter than all the existing LC-UV methods and most LC-MS/MS methods. Besides, no carryover was observed (data not shown).

#### 3.2.2 Lower limit of quantification, calibration curve and range

The LLOQs for 6-TG and 6-MMP were 0.15 μmol/L and 1 μmol/L, respectively, with signals to noise ratio ≥5 (Fig. 2). The LLOQs are lower than the reference concentration ranges in human red blood cells under standard azathioprine regimens, which is sensitive enough to determine the concentrations of 6-TG and 6-MMP for routine TDM. The mean values for the regression equations were *y*_*1*_ = 0.258*x*_*1*_-0.00185 (*r*^2^ = 0.9999) for 6-TG from 0.15 to 15 μmol/L, and *y*_*2*_ = 0.249*x*_*2*_+0.0328 (*r*^2^ = 0.9998) for 6-MMP from 1 to 100 μmol/L. *Y*_*1*_ represented the peak area ratio of 6-TG to IS with *x*_*1*_ representing the concentration of 6-TG in μmol/L. *Y*_*2*_ represented the peak area ratio of 6-MMP to IS with *x*_*2*_ representing the concentration of 6-MMP in μmol/L. The RE between the measured and nominal concentrations of the calibration standards were within ±5%.

#### 3.2.3 Accuracy and precision

The accuracy and precision of within-run and between-run are listed in Table 2. As shown, the CV of within-run for 6-TG and 6-MMP were between 0.5% and 2.4%, and RE ranged from −2.3% to 2.5%. The CV of between-run for 6-TG and 6-MMP were between 1.1% and 3.4%, and RE ranged from −2.6% and 1.8%. The results indicated that this method provides good accuracy and precision.

**Table 2:** Accuracy and precision for 6-TG and 6-MMP.

Analyte	Nominal concentration (μmol/L)	Within-run (*n* = 6)			Between-run (*n* = 18)		
		Mean ± SD	CV (%)	RE (%)	Mean ± SD	CV (%)	RE (%)
6-TG	0.15	0.147 ± 0.004	2.4	−2.0	0.146 ± 0.005	3.4	−2.6
	0.45	0.452 ± 0.009	2.1	0.4	0.453 ± 0.009	1.9	0.7
	3	2.989 ± 0.047	1.6	−0.4	2.996 ± 0.030	1.1	−0.1
	11.25	11.178 ± 0.059	0.5	−0.6	11.201 ± 0.150	1.3	−0.4
6-MMP	1	1.004 ± 0.012	1.2	0.4	0.999 ± 0.019	1.9	−0.1
	3	3.074 ± 0.063	2.0	2.5	3.055 ± 0.047	1.5	1.8
	20	19.976 ± 0.289	1.4	−0.1	20.03 ± 0.376	1.9	0.2
	100	73.247 ± 1.043	1.4	−2.3	73.649 ± 1.666	2.3	−1.8

#### 3.2.4 Recovery

The efficiency of extraction procedure for 6-TG, 6-MMP, and 5-bromouracil in erythrocyte lysate at three concentration levels was listed in Table 3. The extraction recovery in erythrocyte lysate ranged from 53.7% to 54.2% for 6-TG and 94.6%–96.4% for 6-MMP, and the CV was <1.5%. The extraction recovery of the IS in erythrocyte lysate was 87.0% with the CV of 1.5%. These results indicated that the recovery of two analytes and the IS were reproducible and consistent.

**Table 3:** Extraction recovery for two analytes and the internal standard.

Analyte	Nominal concentration (μmol/L)	Extraction recovery (%)	CV (%)
6-TG	0.45	54.2 ± 0.6	1.1
	3	53.7 ± 0.3	0.6
	11.25	53.8 ± 0.2	0.4
6-MMP	3	94.6 ± 1.0	1.1
	20	96.4 ± 0.7	0.7
	75	94.9 ± 0.6	0.7
IS	38.4	87.0 ± 1.3	1.5

#### 3.2.5 Stability

As shown in Table 4, no obvious degradation of 6-TG and 6-MMP was observed in erythrocyte lysate sample after storage at 4 °C for 24 h (RE from −1.3% to 8.0%), at room temperature for 4 h (RE from −4.4% to 3.8%), at −80 °C for 7 d (RE from −3.8% to 9.3%), three freeze-thaw cycles (RE from −1.6% to 3.4%), and post-treatment storage in autosampler (25 °C) for 24 h (RE from −2.2% to 0.7%), within ±15% deviation from the nominal concentrations. Besides, stock solutions of 6-TG and 6-MMP were stable at −80 °C for 30 d within ±6% deviation from the fresh stock solution.

**Table 4:** Stability results for two analytes under different conditions (*n* = 3).

Analyte	Nominal concentration (μmol/L)	Before treatment at 4 °C, 24 h		Before treatment at room temperature, 4 h		Before treatment at −80 °C, 7 d		Three freeze-thaw cycles		Post-treatment in autosampler (25 °C), 24 h	
		Mean ± SD	RE (%)	Mean ± SD	RE (%)	Mean ± SD	RE (%)	Mean ± SD	RE (%)	Mean ± SD	RE (%)
6-TG	0.45	0.486 ± 0.014	8.0	0.467 ± 0.002	3.8	0.492 ± 0.013	9.3	0.446 ± 0.012	−0.9	0.453 ± 0.009	0.7
	11.25	11.104 ± 0.124	−1.3	11.092 ± 0.259	−1.4	12.082 ± 0.124	7.4	11.070 ± 0.169	−1.6	11.059 ± 0.225	−1.7
6-MMP	3	3.072 ± 0.066	2.4	3.057 ± 0.060	1.9	2.901 ± 0.015	−3.3	3.033 ± 0.027	1.1	3.003 ± 0.048	0.1
	75	74.325 ± 1.05	−0.9	71.7 ± 0.375	−4.4	72.15 ± 0.525	−3.8	77.55 ± 1.8	3.4	73.35 ± 1.2	−2.2

A few published data are available regarding the stability of 6-TGN and 6-MMPr sample. In one study, 6-TG concentrations decreased to 53% (at ambient temperature, 22 °C) and 90% (refrigeration, 4 °C) after storage for 7 d, and 6-MMP concentrations decreased to 55% and 86%, respectively [32]. In another, 6-TG and 6-MMP were stable at 25 °C for 4 h and at 4 °C for 24 h before treatment [33], which was consistent with our results.

### 3.3 Method application

After validation, this method is currently applied in one clinical study investigating the relation between concentrations of azathioprine metabolites in red blood cells and the efficacy/adverse reactions of azathioprine in IBD patients. This study was approved by the Ethics Committee of Tongji Hospital (approval number TJ-IRB20200620). We have determined ten clinical samples from ten IBD patients (Table 5) and no interfering peaks were observed.

**Table 5:** Characteristics and measured concentrations of 6-TGN and 6-MMPr in red blood lysate obtained from IBD patients receiving azathioprine therapy.

Patient	Sex	Age (years)	Weight (kg)	Dose (mg/d)	Concentration (pmol/8 × 10^8^RBC)	
					6- TGN	6-MMPr
1	male	31	61	50	113.6	100.5
2	female	45	50	50	179.3	980.2
3	female	28	49	75	332.5	1650.8
4	male	17	62	75	424.7	1998.5
5	male	24	70	75	336.8	220.7
6	male	39	76	100	300.6	156.8
7	male	35	75	100	521.5	1489.6
8	female	47	62	100	297.6	3778.6
9	male	26	67	100	240.4	280.8
10	female	45	63	150	537.4	3336.8

## 4 Conclusion

A rapid, specific, and accurate HPLC-UV method for simultaneous determination of azathioprine metabolites 6-TGN and 6-MMPr in human red blood lysate was developed in this work. This novel method is marked with its rapid turn-around time of 5.5 min, which is shorter than all the reported LC-UV methods and most of the LC-MS/MS methods, and less mobile phase solution consumption. Besides, this assay exhibited good sensitivity and stability, and could be employed in routine TDM of azathioprine for IBD patients.

## Author contribution statement

Dong Liu and Xuepeng Gong conceived and designed the experiments; Hengyi Yu performed the experiments; Dongyan Li and Dong Xiang analyzed and interpreted the data; Xiping Li and Lu Liu contributed reagents, materials and analysis tools; Hengyi Yu, Dong Liu and Xuepeng Gong wrote the paper.

## Conflicts of competing interest

There are no conflicts to declare.
